# Association between Severe Dehydration in Rotavirus Diarrhea and Exclusive Breastfeeding among Infants at Dr. Hasan Sadikin General Hospital, Bandung, Indonesia

**DOI:** 10.1155/2015/862578

**Published:** 2015-11-03

**Authors:** Dwi Prasetyo, Iesje Martiza Sabaroedin, Yudith Setiati Ermaya, Yati Soenarto

**Affiliations:** ^1^Department of Child Health, Faculty of Medicine, Universitas Padjadjaran, Dr. Hasan Sadikin General Hospital, Bandung 40161, Indonesia; ^2^Departments of Child Health and Microbiology, Faculty of Medicine, Universitas Gadjah Mada, Sardjito Hospital, Yogyakarta 55281, Indonesia

## Abstract

*Background*. Rotavirus is the leading cause of severe acute diarrhea in children. Infants who are exclusively breastfed develop fewer infections and have less severe illnesses. This study aimed to determine association between severe dehydration in rotavirus diarrhea and exclusive breastfeeding. * Methods*. This is a cross-sectional study in infants ≤ 6 months old with acute diarrhea in Dr. Hasan Sadikin Hospital, Bandung, Indonesia. * Results*. From 134 infants ≤ 6 months old with acute diarrhea enrolled from April 2009 to December 2012, there were 88 (65.6%) boys and 46 (34.4%) girls in this study. Rotavirus was detected in 60 (44.8 %), 32 (53.3%) of whom were exclusively breastfed. From rotavirus positive subjects, severe dehydration occurred in 4 (12.6%) exclusively breastfed infants and 6 (21.5%) not exclusively breastfed infants. No significant association was found between severe dehydration and exclusive breastfeeding (*p* = 0.491) in rotavirus diarrhea. *Conclusions*. In rotavirus diarrhea, there was no significant association between exclusive breastfeeding and severe dehydration.

## 1. Background

The main cause of diarrhea in children worldwide is rotavirus [1]. In recent years, rotavirus has been recognized as one of the most common causes of diarrhea, both in developed and in developing countries [1]. Globally, in 2008, rotavirus diarrhea is estimated to cause 453,000 deaths (95% CI, 420,000–494,000) in children younger than 5 years, contributing to 37% of all deaths due to acute diarrhea [2].

Rotavirus was isolated in 30–73% of diarrhea cases in children in the Asian region [1]. In Thailand, from 2001 to 2003, community surveillance showed the proportion of cases of rotavirus diarrhea in the community to be much lower than that in the hospitalized population (12.2% versus 43%) [3]. In Hong Kong and Iran, the rate of rotavirus diarrhea in children under 5 years of age was 30% [4] and 59.1% [5], respectively. In 2003, 39.8% of children under five years of age with acute gastroenteritis admitted to three pediatric hospitals in Turkey [6]. In Indonesia, the rate of rotavirus diarrhea in under-five inpatients was 37–69%, while a 40% rate was seen among outpatients [7]. In Bandung, the prevalence of rotavirus in hospitalized children was 47% in 2006 [8].

Breast milk contains nutrients, antioxidants, hormones, and antibodies needed by a child to survive and develop [9]. Infants who are exclusively breastfed during the first 6 months of life and continue to be breastfed until two years of age develop fewer infections and less severe illnesses [9]. Infants who are not breastfed have a sixfold greater risk of dying from infectious disease in the first two months of life, including from diarrhea [9]. Breastfeeding could reduce gastrointestinal infections as breast milk contains lactadherin, secretory IgA, T and B lymphocyte, bactericidal lactoferrin, oligosaccharides, and human milk glycans [10, 11]. Although the anti-rotavirus antibodies in human milk play a small role, the main component of human milk that prevents rotavirus infection seems to be lactadherin [12].

There is consistent evidence of a protective effect against diarrhea from exclusive breastfeeding in the first 4–6 months of life [13]. The relationship is not consistent for rotavirus infections but it is consistently strong for nonviral pathogens [13]. However, other study did not reveal breastfeeding as protective against rotavirus diarrhea in infants [10].

This study aimed to determine association between severe dehydration in rotavirus diarrhea and exclusive breastfeeding.

## 2. Methods

This is a prospective cross-sectional study. Subjects were infants ≤ 6 months old with acute diarrhea admitted to Dr. Hasan Sadikin General Hospital, Bandung (referral hospital of West Java Province, Indonesia), who were enrolled by consecutive sampling from April 2009 to December 2012. Infants with bloody diarrhea were excluded. Fresh fecal specimens were obtained from all the subjects within 24 hours after admission and stored at 20°C before rotavirus detection was performed in the laboratory of microbiology. Rotavirus was tested by Rota Antigen Detection using ProSpecT Rotavirus Microplate Assay according to the standard operating procedures.

Information on breastfeeding was obtained from questionnaire. Exclusive breastfeeding was defined as no food or drink, not even water, other than breast milk given to infants for 6 months of life [14]. However, it allows the infant to receive oral rehydration solution, drops, and syrups (vitamins, minerals, and medicines) [14].

The definition of “diarrhea” in this study was the passage of at least three loose or watery stools in a 24-hour period, based on the World Health Organization (WHO) definition [15]. The dehydration state was classified based on the WHO guideline of no sign of dehydration, some dehydration, and severe dehydration [15].

### 2.1. Statistical Analysis

Data were expressed as sums and percentages. Baseline characteristics of rotavirus diarrhea dehydration and breastfeeding were compared using Epi Info v.3.5.4 with a *p* value of <0.05 were considered significant. Categorical data were analyzed by Chi-squared test and exact Fisher test.

## 3. Results

There were 134 subjects ≤ 6 months old, 88 (65.6%) boys and 46 (34.4%) girls, with acute diarrhea in this study.

This study showed that exclusive breastfeeding had no significant association with sex, maternal occupation, maternal education, and nutritional assessment of child, with *p* = 0.062, 0.666, 0.191, and 0.896, respectively (Table 1).

We found 24 (17.9%) subjects with severe dehydration. Rotavirus was positively detected in 60 (44.8%) from all subjects (Table 2).

From rotavirus positive samples, 32 (53.3%) were exclusively breastfed. From rotavirus positive subjects, severe dehydration occurred in 4 (12.6%) exclusively breastfed infants and 6 (21.5%) not exclusively breastfed infants. No significant association was found between severe dehydration and exclusive breastfeeding (*p* = 0.491) in rotavirus diarrhea (Table 3).

## 4. Discussion

From April 2009 to December 2012, 134 infants ≤ 6 months with diarrhea admitted to Dr. Hasan Sadikin General Hospital were recruited for this study. We found that 60 (44.8%) samples were infected by rotavirus. This result is similar to other studies, such as a study by Nakawesi et al. in Uganda (45.4%) [16] and studies in several countries in the Asian region (estimated 30%–70%) [2]. A previous study in Indonesia by Soenarto et al. also found 39%–67% rotavirus diarrhea cases [7].

This study showed that, in rotavirus positive subjects, severe dehydration occurred in 4 (12.6%) exclusively breastfed infants and 6 (21.5%) not exclusively breastfed infants. We found that the severe dehydration rotavirus diarrhea in infants ≤ 6 months old who received exclusive breastfeeding was not significantly different from those who did not receive exclusive breastfeeding. Previous studies have shown similar results, including a study by Wobudeya et al. in Uganda that claimed exclusive breastfeeding does not offer protection against rotavirus diarrhea [10]. In a study by Linhares et al. in Brazil among children aged 0–3 years who suffered from rotavirus diarrhea, breastfeeding is proven as unable to protect children from rotavirus diarrhea [17]. In India, there is a high incidence of rotavirus infection but exclusive breastfeeding cannot provide enough protection to infants [18].

Studies showed different results in the relationship between exclusive breastfeeding and rotavirus diarrhea, not all showing benefits from exclusive breastfeeding in reducing the severity. The specific role of breastfeeding in the prevention of rotavirus diarrhea has not been well established; however, it is generally considered that it at least reduces the severity of the disease. Several previous studies that support the positive influence of exclusive breastfeeding on the incidence of diarrhea include a study in Malaysia by Prameela and Vijaya that claimed a relationship between breastfeeding and protection against rotavirus diarrhea [19]. Studies in Germany, Switzerland, and Austria that assess whether breastfeeding infants in the community (community-based) aged 0–12 months gain protection against rotavirus diarrhea showed that exclusively breastfed infants may have reduced risk of diarrhea caused by rotavirus. In infants aged 0–6 months, the protective effect is stronger than in infants aged 7–12 months [20]. A study in Mexico by Sánchez-Uribe et al. in children aged <36 months with rotavirus diarrhea showed that exclusively breastfed infants have a positive significant association with reduced risk of diarrhea caused by rotavirus (*p* < 0.01) [21]. A study in the United States by Dennehy et al. that aimed to determine the risk factors for the occurrence of rotavirus diarrhea in hospital among children aged <59 months showed a protective effect in infants aged <6 months who are exclusively breastfed in terms of the incidence of rotavirus diarrhea leading to hospitalization [22].

Exclusive breastfeeding is found to protect infants from severe rotavirus diarrhea in Bangladesh, according to a study by Clemens et al. in 1993 [23]. Another study in Brazil on human breast milk has shown the presence of secretory IgA antibodies and rotavirus G9P(5) neutralizing capacity. A strong correlation is seen between the level of anti-rotavirus antibody and the neutralizing capacity of breast milk samples, supporting the possible role of this antibody in protecting against infection [24].

Without disregarding the efforts to promote breastfeeding to reduce the burden of diarrhea in general, our findings showed that breastfeeding does not provide protection against severe dehydration in rotavirus diarrhea.

The findings of this study suggest that another preventive method against rotavirus diarrhea, such as rotavirus vaccination, is needed. The World Health Organization has recommended that rotavirus vaccination is included in all national immunization programs to provide protection against rotavirus diarrhea. However, only a few, mostly high- and middle-income, countries include rotavirus vaccine in their immunization schedules [9]. Currently, rotavirus immunization in Indonesia, including West Java, is not yet included in the national program. This is due to the fact that the cost of available vaccines is high; thus only a small portion of citizens could afford it. Unfortunately there are insufficient data regarding rotavirus vaccine coverage in Indonesia.

## 5. Conclusion

In rotavirus diarrhea, there was no significant association between exclusive breastfeeding and severe dehydration.

## Figures and Tables

**Table 1:** Exclusive breastfeeding based on sociodemographic characteristics.

Characteristics	Total (%)	Exclusive breastfeeding		*p* value^*∗*^
		Yes	No	
(1) Sex				
Male	88 (65.6)	45	43	0.062
Female	46 (34.4)	15	31	
(2) Maternal occupation				
Worker	21 (15.6)	8	13	0.666
Housewife	113 (84.4)	52	61	
(3) Maternal education				
No education	1 (0.7)	1	0	0.191
Elementary	34 (25.5)	11	23	
Junior high school	47 (35.0)	27	20	
Senior high school	42 (31.4)	18	24	
Diploma	4 (2.9)	1	3	
Bachelor	6 (4.5)	2	4	
(4) Nutritional assessment of child				
Good	99 (73.9)	45	54	0.896
Mild malnutrition	29 (21.6)	12	17	
Severe malnutrition	6 (4.5)	3	3	

^*∗*^Chi-squared test.

**Table 2:** Dehydration degree and rotavirus in infants ≤ 6 months of age.

Variable	Number (%)
(1) Dehydration degree	
No dehydration	23 (17.2)
Some dehydration	87 (64.9)
Severe dehydration	24 (17.9)
(2) Rotavirus	
Positive	60 (44.8)
Negative	74 (55.2)

**Table 3:** Association between severe dehydration and exclusive breastfeeding in infants with rotavirus diarrhea.

Dehydration degree (rotavirus positive)	Exclusive breastfeeding		*p* value
	Yes *n* (%)	No *n* (%)	
No dehydration	3 (9.3)	5 (17.8)	0.454^*∗∗*^
Some dehydration	25 (78.1)	17 (60.7)	0.236^*∗*^
Severe dehydration	4 (12.6)	6 (21.5)	0.491^*∗∗*^

^**∗**^Chi-squared test; ^*∗∗*^exact Fisher test.
